# MRI findings in cervical spondylotic myelopathy with gadolinium enhancement: Review of seven cases

**DOI:** 10.1259/bjrcr.20200133

**Published:** 2021-01-05

**Authors:** Lucas María Pessini Ferreira, Cristina Auger, Izaro Kortazar Zubizarreta, Gonzalo Gonzalez Chinchon, Isabel Herrera, Albert Pla, Andrea de Barros, Carlos Tortajada, Alex Rovira

**Affiliations:** 1 Hospital Universitario Vall d'Hebron, Barcelona, Spain; 2 Hospital Universitario Fundación Jiménez Díaz, Madrid, Spain; 3 Hospital General Universitario Gregorio Marañón, Madrid, Spain

## Abstract

Cervical spondylotic myelopathy (CSM) is a clinical syndrome secondary to a spinal cord compression due to cervical spondylosis. In some cases, conventional MRI typically shows an intramedullary hyperintense signal on T2W imaging and contrast enhancement on post-gadolinium T1W imaging. We report a series of seven patients with CSM who had typical clinical presentation and imaging findings on T2W and contrast-enhanced T1W sequences. The imaging findings included degenerative changes of the cervical spine, intramedullary T2-signal hyperintensity, and an intramedullary enhancement on post-gadolinium T1W images. Our results support the statement that the presence of an intramedullary gadolinium-enhancement with a flat transverse pancake-like pattern (on sagittal images) and a circumferential pattern (on axial images), located within a T2-signal abnormality, in patients with cervical spondylosis and clinical myelopathy is indicative of spondylosis as the cause of the myelopathy.

CSM consists of a clinical syndrome secondary to a spinal cord compression due to cervical spondylosis.^1,2^ This is the most common cause of spinal cord dysfunction worldwide. The pathophysiology of CSM is multifactorial and results from the accumulation of cervical spinal degenerative changes,^3^ which leads to a narrowing of the spinal canal diameter and a loss of its sagittal mobility.^4^ The setting of a congenital cervical spinal stenosis may increase the risk of developing CSM.^5^

The clinical presentation of CSM is typically insidious and may include gait impairment, inferior limbs numbness and loss of hand dexterity.^6^ The natural history of CSM may be mixed: many patients experience a slow decline, others experience periods of quiescence, and a subgroup of patients may improve.^7^

Evidence from both postmortem histological^8^ and *in vivo* imaging^9^ studies demonstrate that demyelination occurs in patients with CSM. If a severe stenosis persists over time, patients may also develop necrosis of both gray and white matter.^7^

In some cases of CSM, MRI typically show intramedullary signal abnormalities due to edema and structural changes with a hyperintense signal on T2W imaging (WI) (58–85% of patients with clinical myelopathy) and, less commonly, hypointense signal on T1-WI.^10^

Although post-gadolinium MRI sequences are not routinely used in the assessment of cervical degenerative disease, they could provide information about the integrity of the spinal cord parenchyma^11^ and its blood-spinal cord barrier: intramedullary contrast enhancement would indicate local areas of disruption of the blood-spinal cord barrier of white matter vessels, most likely venous channels.^12^ In patients with clinical myelopathy, a pancake-like gadolinium enhancement on sagittal images and a circumferential enhancement on axial images are indicative of CSM.^13–15^ Here, we report a series of seven patients who had the typical MRI findings of CSM on post-contrast T1-WI.

## Case series

Seven cases of CSM with enhancement after gadolinium administration were retrospectively identified from three different centers (mean age, 51). Written informed consent was obtained from the patients for publication of this case review, including accompanying images

**Clinical manifestations** (Table 1): the onset was insidious (more than 8 weeks) in six patients and subacute (less than 8 weeks) in one. All our patients experienced weakness and paresthesias, six had numbness and two had pain. The youngest (aged 38) had bladder dysfunction and was also the only one with a subacute onset.

**Table 1.** Clinical features of our case series

Case	Age (years)	Sex	Clinical onset		Clinical features				
			Subacute	Insidious	Numbness	Paresthesia	Pain	Weakness	Bowel/bladder
1	39	M	No	Yes	Yes	Yes	No	Yes	No
2	59	M	No	Yes	No	Yes	No	Yes	No
3	52	F	No	Yes	Yes	Yes	Yes	Yes	No
4	45	M	No	Yes	Yes	Yes	No	Yes	No
5	38	M	Yes	No	Yes	Yes	No	Yes	Yes
6	62	F	No	Yes	Yes	Yes	Yes	Yes	No
7	62	F	No	Yes	Yes	Yes	No	Yes	No
Percentage (%)	-	-	14.3	85.7	85.7	100	28.6	100	14.3

**Figure 1.**
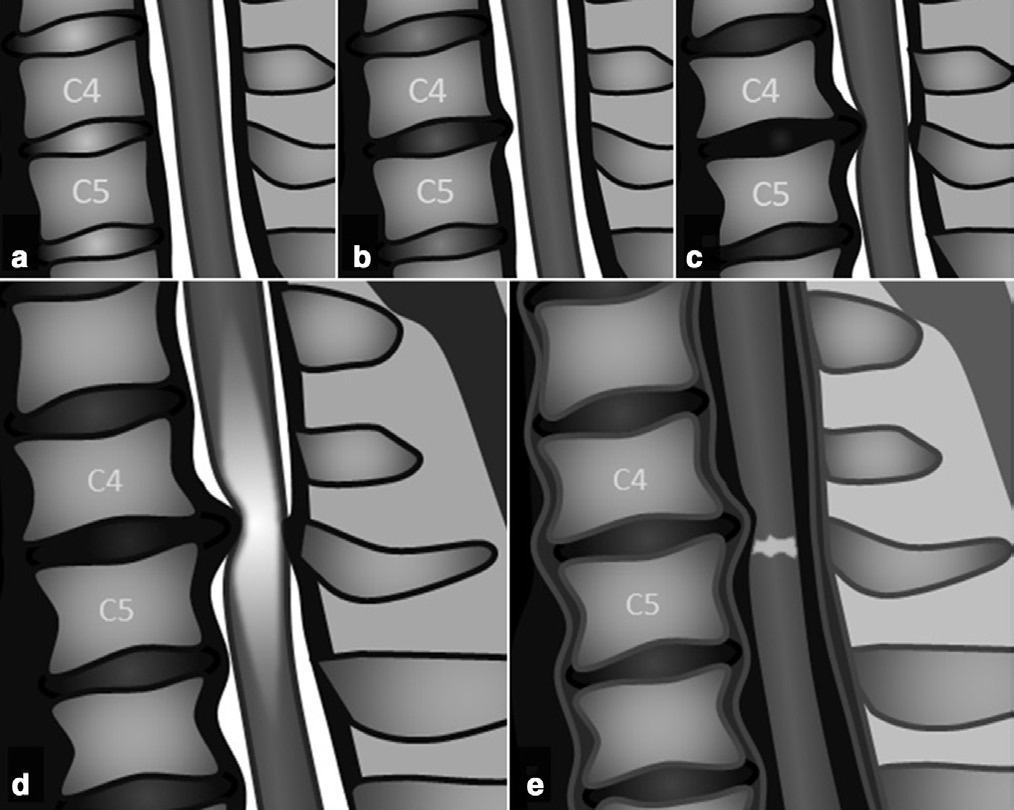
Schematic representation of the MRI Grading System proposed by Kang et al^16^ on sagittal T2-WI. Grade 0, absence of canal stenosis (A); Grade 1, subarachnoid space obliteration exceeding 50% (B); Grade 2, spinal cord deformity (C); Grade 3, spinal cord signal change (D shows T2-signal change, and E shows contrast enhancement on gadolinium-enhanced T1-WI imaging).

**Imaging findings** (Tables 2 and 3): They included cervical spondylotic changes and degenerative disc disease, intramedullary T2-signal hyperintensities, and intramedullary enhancement on post-gadolinium T1-WI. The severity of cervical stenosis was classified following the MRI Grading System proposed by Kang et al.^16^ (Figure 1, Table 4). All seven patients had a Grade 3 cervical stenosis, being more prevalent at C4-C5.

**Table 2.** Imaging findings on *T*_2_-weighted magnetic resonance imaging sequences

Case	Level of maximum stenosis	T2-WI Signal abnormality	Sagittal T2-WI				Axial T2-WI	
			Extension		Shape		Signal at Gd-enhancement site	
			>3 VB	<3 VB	Spindle	Focal	Heterog.	Central location
1	C6-C7	Yes	Yes	No	Yes	No	Yes	Yes
2	C4-C5	Yes	No	Yes	No	Yes	No	Yes
3	C3-C4	Yes	Yes	No	Yes	No	Yes	Yes
4	C4-C5	Yes	No	Yes	Yes	No	Yes	Yes
5	C5-C6	Yes	Yes	No	Yes	No	Yes	Yes
6	C5-C6	Yes	Yes	No	Yes	No	No	Yes
7	C4-C5	Yes	No	Yes	No	Yes	Yes	Yes
Percentage (%)	-	100	57.1	42.9	71.4	28.6	71.4	100

Gd, Gadolinium; Heterog, Heterogeneous; VB, Vertebral Bodies; WI, weighted imaging.

**Table 3.** Imaging findings on post-contrast *T*_1_-weighted MRI sequences

Case	Level of maximum stenosis	Sagittal T1-WI Gd-enhancement			Axial T1-WI Gd-enhancement			
		Transverse band (pancake-like)		Focal enhancement	Circum.	Focal	Patchy	Diffuse
		Complete	Central- sparing					
1	C6-C7	Yes	No	No	Yes	No	No	No
2	C4-C5	No	No	Yes	No	Yes	No	No
3	C3-C4	Yes	No	No	No	No	No	Yes
4	C4-C5	Yes	No	No	Yes	No	No	No
5	C5-C6	No	No	Yes	Yes	No	No	No
6	C5-C6	No	No	Yes	Yes	No	No	No
7	C4-C5	No	Yes	No	Yes	No	No	No
Percentage (%)	-	42.9	14.3	42.9	71.4	14.3	0	14.3

Circum, Circumferential; Gd, Gadolinium; WI, weighted imaging.

**Table 4.** Distribution of stenoses grading (Kang et al) by cervical level

Cervical level	Grade 0	Grade 1	Grade 2	Grade 3
C2-C3	5	2	0	0
C3-C4	2	4	0	1
C4-C5	1	1	2	3
C5-C6	0	1	4	2
C6-C7	0	5	1	1
C7-D1	4	2	1	0

Grade 0, absence of canal stenosis; Grade 1, subarachnoid space obliteration; Grade 2, spinal cord deformity; Grade 3, spinal cord signal change.

**Figure 2.**
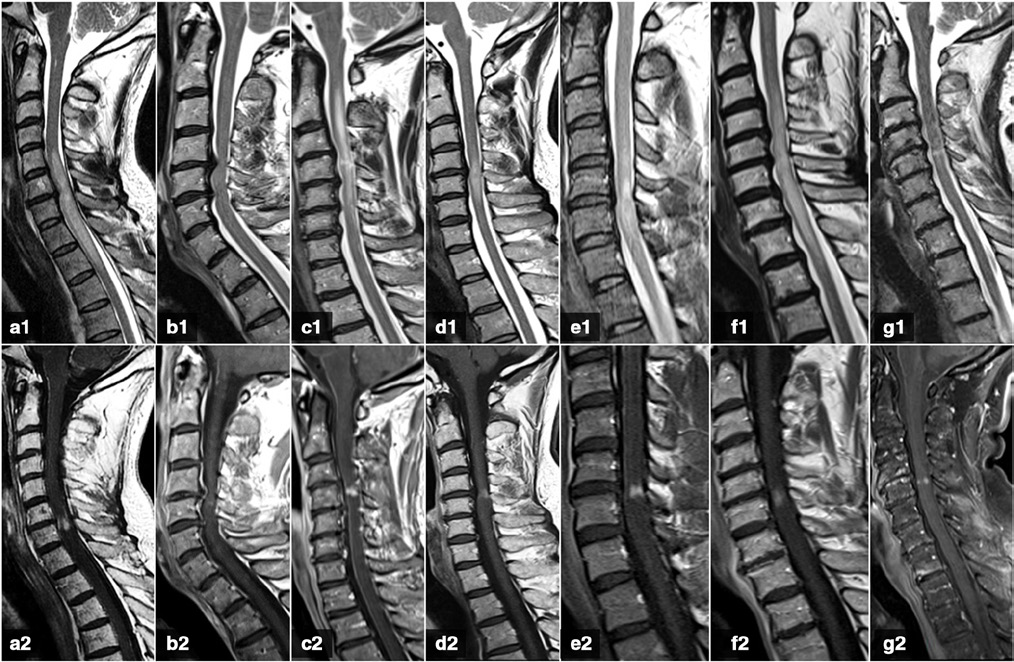
First MRI scans from our case series, sagittal plane. Sagittal T2-WI (A1-G1) and post-contrast T1-WI (A2-G2) show intramedullary spindle-shaped T2-signal hyperintensities in cases 1, 3, 4, 5 and 6 (A1, C1-F1) and focal-shaped in cases 2 and 7 (B1, G1). Also, gadolinium enhancement is seen as a transverse band in cases 1, 3, 4 and 7 (A2, C2, D2, G2); and a focal enhancement in cases 2, 5 and 6 (B2, E2, F2).

On T2-WI sequences (Figure 2, Table 2), all our patients had an intramedullary hyperintense T2-signal area. Its longitudinal extension was greater than the height of three vertebral bodies in four patients (57%,) and smaller in the other three (43%). This area had a spindle-like shape in five patients and a focal-shape in the other two. A T2-signal heterogeneity was detected at the site of the enhancement in five cases. In the axial plane, the T2-signal hyperintensity was centrally-located in all the patients. We found no cases with associated syrinx or cystic changes within the spinal cord.

**Figure 3.**
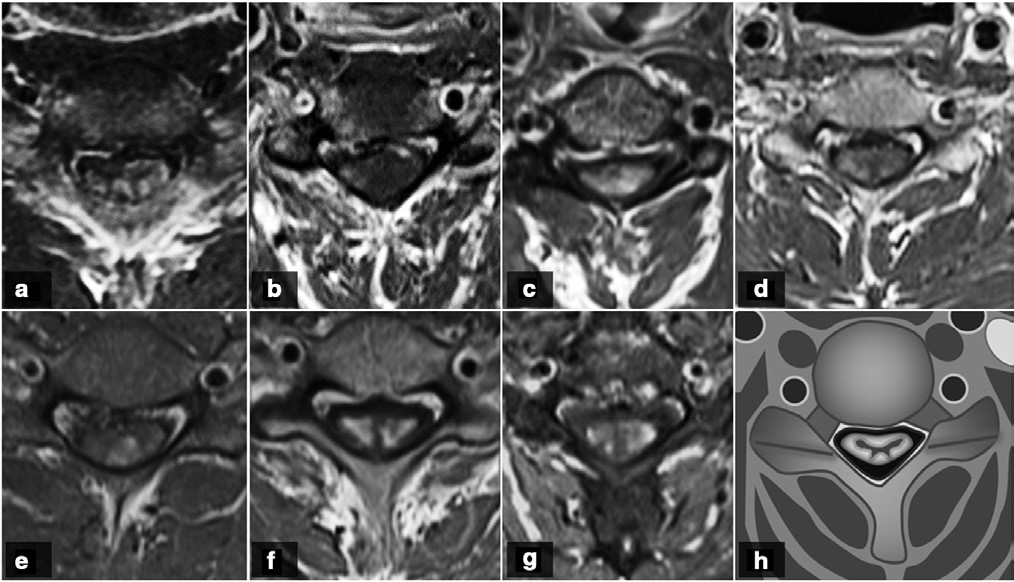
First magnetic resonance imaging scans from our case series, axial plane. Axial gadolinium-enhanced T1-WI showing a circumferential enhancement in cases 1 and 4–7 (A, D, E, F, G), focal in case 2 (B) and diffuse in case 3 (C). A schematic representation of a circumferential enhancement on post-contrast T1-WI is shown, sparing the central gray matter (H).

As for post-contrast T1-WI sequences (Figure 2, Table 3), all our patients showed intramedullary gadolinium enhancement, immediately below the site of maximum stenosis (Grade 3), being C4-C5 the most prevalent location. Sagittal images showed an intramedullary flat transverse band of enhancement in four patients. This band was complete in three patients and incomplete (central sparing) in one. The other three patients showed a focal enhancement. In the axial plane (Figure 3), the enhancement was circumferential in four cases, being focal, multifocal and diffuse in the remaining three (one of each type), always sparing the central gray matter.

### Treatment

Five patients received initial treatment with i.v. methylprednisolone, and four underwent a decompressive surgery at the level of maximum cervical stenosis (Table 5). The mean interval from diagnostic MRI to surgery was 3.2 months (range 2–5).

**Table 5.** Treatment and follow-up MRI

Case	Surgery		IVMP	Clinical follow-up cry		Radiological follow-up		
	Performed	Time^a^		Time^a^	Evolution	Time^a^	T2-signal hyperintensity	Gd T1-WI enhancement
1	Nob	-	Yes	12	Partial recovery	12	Persisted, atrophy	Persisted
2	Yes	2	No	12	Significant recovery	NA	NA	NA
3	Nob	-	No	48	Slow progression	48	Mild improvement	Mild improvement
4	Yes	5	Yes	30	Partial recovery	20	Improvement	Resolution
5	Yes	3	Yes	15	Partial recovery	12	Mild improvement	Mild improvement
6	Yes	3	Yes	6	Significant recovery	4	Persisted	NA
7	Nob	-	Yes	36	Slow progression	36	Persisted, atrophy	NA

IVMP, intravenous methylprednisolone; NA, not available.

a Time is expressed in months from the first magnetic resonance imaging scan

b The patient refused undergoing surgical treatment

### Clinical follow-up

The four operated patients improved (significantly in two cases and partially in the other two). Among the three patients who refused surgical treatment, one had a mild partial improvement and the other two showed a slow clinical progression.

### Imaging follow-up

**Figure 4.**
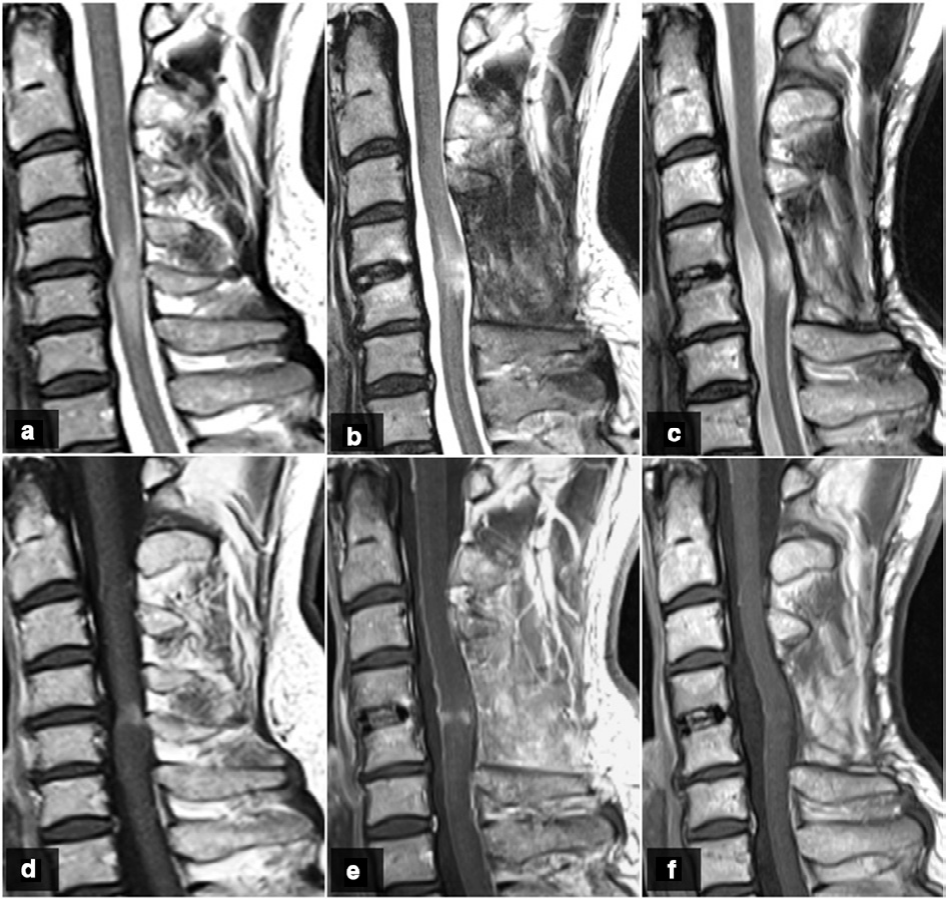
Scans from case 4. Pre-surgical scan: stenosis at C4-C5 with CSM (A) and contrast enhancement (D). One month after surgery: partial improvement of T2-signal hyperintensity (B) and partial resolution of contrast enhancement (E). One year after surgery: no changes in the T2-signal hyperintensity (C) and a complete resolution of enhancement (F).

One patient had no MRI follow-up after surgery due to a complete clinical resolution. The other six (three operated and three non-operated) were radiologically followed up to an average of 22 months. Among the **operated** patients, the spinal T2-signal changes improved in two cases and persisted in one. Gadolinium was administered in two operated patients, showing a complete resolution of the enhancement in one case and a mild improvement in the other (Figure 4). Regarding the **non-operated** patients, the spinal T2-signal changes persisted in two cases, and improved in the other one. Gadolinium was administered in two non-operated patients, showing a persistence of the enhancement in one and an improvement in the other.

## Discussion

All our patients presented intramedullary T2-signal abnormalities, which is a constant finding on every similar reported cases.^11,13–15,17–19^ Regarding its longitudinal extension, it was greater than the height of three vertebral bodies in 57% of our patients. These results slightly differ from those obtained by *Flanagan et al*. (45%, *N* = 56)^14^ but are closer to those obtained by Ozawa et al. (52%, *N* = 50),^17^ without losing sight of the potential bias due to a relatively small sample size of our series (*N* = 7). On sagittal images, its morphology was spindle-like shaped in 71% of our patients, which has been described as a typical finding.^13–15^ A focal T2-signal heterogeneity was detected in coincidence with the site of contrast enhancement in 71% of our patients, a finding that has been previously reported in 23%.^14^ This difference may also be due to a sample size-related bias. None of our patients had associated syrinx or cystic changes within the spinal cord on T2-WI sequences.

Gadolinium enhancement in CSM has been mentioned in previously published case series^11,17,18^ and single-case reports,^13,15,19^ but we highlight the contributions of Flanagan et al^14^ in describing in full-detail the specific pattern of enhancement.

On post-contrast sagittal images, intramedullary flat pancake-like enhancement was seen in 57% of our patients, compared with 73% reported in the Flanagan et al series.^14^ This transverse band of enhancement was complete in 43% and incomplete in 14% in our series, compared with 67 and 6%, respectively, reported by the same authors.^14^ The longitudinal extension of the enhancement was less than the height of one vertebral body, with a transverse diameter always larger than the longitudinal diameter. On post-contrast axial images, a circumferential enhancement pattern was detected in 71.4% of our patients, compared with 57% also reported by Flanagan et al and 46% by Ozawa et al..^14,17^ No cases of patchy enhancement were detected in our series, compared with a 14 and 30% reported by the same authors.

Other potential causes of spinal cord enhancement should be considered in the differential diagnosis, including demyelinating, metabolic, neoplastic, and vascular etiologies.^20^ A systematic approach to the enhancement pattern and other imaging features will allow for a more specific interpretation of scan results.^20^ Acute myelopathies resulting from infectious agents should also be considered in the differential diagnosis, although in these cases fever, meningismus, and inflammatory CSF usually lead to investigation of a causative agent.^21^

Potential alternative diagnoses were proposed in our patients, including spinal cord tumors, transverse myelitis, multiple sclerosis, sarcoidosis and neuromyelitis optica (NMO), but laboratory test results, the non-improvement after steroid therapy, and clinical -radiological follow up disregarded those diagnoses in favor of CSM. The differential diagnosis with spinal cord tumors was particularly difficult in two cases (cases 3 and 4). A fluorine-18 fluorodeoxyglucose (FDG) positron emission tomography/computed tomography (PET/CT) was performed in one of these patients (case 4), showing a hypermetabolic focus within the contrast-enhancement area. This gave the impression of a neoplastic disease, which lead to a decompressive surgery, and partial resection of the lesion. There is evidence of one similar case in the literature,^14^ with similar MRI findings and pre-surgical diagnosis, which also turned out to be a pathologically proven CSM.

Among the methodological limitations of this case series, we include the retrospective nature of data collection and its small sample size in comparison with similar series from the literature.^11,14,17^ Despite these limitations, our results support the statement^14,15^ that recognizing a specific pattern of gadolinium-enhancement on sagittal and axial MRI, in coincidence with an intramedullary T2-signal abnormality in patients with cervical spondylosis and clinical myelopathy, is indicative of spondylosis as the cause of myelopathy. Recognizing this radiological pattern may contribute to an accurate diagnosis and to the exclusion of other causes of myelopathy. Therefore, it may help define the treatment strategies avoiding unnecessary interventions.

## Teaching points

- In patients with CSM, a persistent spinal canal stenosis may lead to spinal cord demyelination and also necrosis. Contrast enhancement would indicate local areas of disruption of the blood-spinal cord barrier of white matter vessels, most likely venous channels.
- Differential diagnosis can be challenging, especially when the imaging features resemble those found in spinal neoplastic disease. Special attention should be taken if a FDG PET/CT is performed in order to rule out this diagnosis, as CSM and neoplasms can both show hypermetabolic areas within the spinal cord, in coincidence with the site of contrast enhancement on MRI.
- In patients with clinical myelopathy, a pancake-like gadolinium enhancement on sagittal images and a circumferential enhancement on axial images are indicative of CSM as the cause of myelopathy.
